# Skin Immunomodulation during Regeneration: Emerging New Targets

**DOI:** 10.3390/jpm11020085

**Published:** 2021-01-30

**Authors:** Loubna Mazini, Luc Rochette, Yousra Hamdan, Gabriel Malka

**Affiliations:** 1 Centre des Sciences Biologiques et Médicales (CIAM), Université Mohammed VI Polytechnique, Ben-Guerir 43150, Morocco; yousra.hamdan@um6p.ma (Y.H.); gabriel.malka@um6p.ma (G.M.); 2 Equipe d’Accueil (EA 7460): Physiopathologie et Epidémiologie Cérébro-Cardiovasculaires (PEC2), Faculté des Sciences de Santé, Université de Bourgogne—Franche Comté, 7 Bd Jeanne d’Arc, 21000 Dijon, France; luc.rochette@u-bourgogne.fr

**Keywords:** adipose-derived stem cells, skin, immunomodulation, aging, regeneration, exosomes, biomarkers

## Abstract

Adipose-Derived Stem Cells (ADSC) are present within the hypodermis and are also expected to play a pivotal role in wound healing, immunomodulation, and rejuvenation activities. They orchestrate, through their exosome, the mechanisms associated to cell differentiation, proliferation, and cell migration by upregulating genes implicated in different functions including skin barrier, immunomodulation, cell proliferation, and epidermal regeneration. ADSCs directly interact with their microenvironment and specifically the immune cells, including macrophages and T and B cells, resulting in differential inflammatory and anti-inflammatory mechanisms impacting, in return, ADSCs microenvironment and thus skin function. These useful features of ADSCs are involved in tissue repair, where the required cell proliferation, angiogenesis, and anti-inflammatory responses should occur rapidly in damaged sites. Different pathways involved have been reported such as Growth Differentiation Factor-11 (GDF11), Tumor Growth Factor (TGF)-β, Metalloproteinase (MMP), microRNA, and inflammatory cytokines that might serve as specific biomarkers of their immunomodulating capacity. In this review, we try to highlight ADSCs’ network and explore the potential indicators of their immunomodulatory effect in skin regeneration and aging. Assessment of these biomarkers might be useful and should be considered when designing new clinical therapies using ADSCs or their specific exosomes focusing on their immunomodulation activity.

## 1. Introduction

The skin acts as a protective barrier with its three layers epidermis, dermis, and hypodermis, but its role in water retention, thermoregulation, and cell regeneration is critical. However, it remains the first exhibition of age, visible externally as skin winkle and accompanied by integrity, elasticity, and functionality loss. Aging is differently appreciated between individuals, but similar cellular and molecular changes are involved, resulting in a progressive reduction in skin cell regeneration as the consequence of increasing cell senescence and apoptosis [1].

Adipose Derived Stem Cells (ADSC) are considered Mesenchymal Stem Cells (MSC), or MSC-like, which have a fibroblast-like morphology and are plastic adherent. According to the International Federation of Adipose Tissue and Therapeutics (IFATS) and the International Society for Cellular Therapy (ISCT), ADSCs differentiate into osteogenic, adipogenic, and chondrogenic cell lineages and present the stromal associated markers CD13, CD29, CD44, CD73, CD90, CD105, and CD106 [2,3,4]. These cells are highly proliferative, and their differentiation capacity was maintained with aging [5]. ADSCs are less immunogenic having no human leucocytes antigen-DR (HLA-DR) proteins on their surface and have immunomodulatory effects, making them immunosuppressive effects and suitable for clinical applications at both autologous and allogeneic settings [6,7].

ADSCs are identified within the hypodermis layer and have been reported to commit into skin cells to maintain homeostasis [8,9,10]. Their capacity to self-renew in vitro and in vivo and to differentiate innately opened the way to promising advancements in regenerative medicine by ensuring full-thickness skin replacement [11,12]. Clinical applications of ADSCs benefit from their simple and abundant collection and gain more interest regarding their immunomodulatory effects, favoring their suitable use compared to their counterparts from bone marrow (BM) and umbilical blood (UC) [13,14], especially in autoimmune diseases and inflammatory-associated diseases [8,15,16,17,18,19]. There are evidences that these cells act through cell-cell contact and especially by secreting different biomolecules (growth factors, cytokines, and chemokines, messenger ribonucleic acid (mRNA), non-coding-RNA (nc-miR) such as micro-RNA (miRNAs) and lncRNAs) within extracellular vesicles. These exosomes are involved in different cell biological process in normal and pathological settings [15,20,21,22,23,24,25,26]. Indeed, ADSCs have been reported to regulate inflammation and participate in the phases of wound healing through their exosomes [10,24,25,26,27,28,29]. Within their microenvironment, ADSCs carried their immune-modulatory and regenerative effects through direct interaction with activated immune cells [30,31,32]. This cell network is the key regulator of skin regeneration and repair. Therefore, ADSCs can be differentiated towards the desired cell phenotype as a result of microenvironment stimulation or the appropriate composition of cell culture media. This cell priming suggests that ADSCs’ behaviors and interactions within their microenvironment would condition the exosomes ‘composition, cell immunomodulation, and thus tissue regeneration. These exosomes represent the signaling pathways between and within cells, thus promoting and accelerating skin regeneration [10,24,26,33,34,35]. Actually, exosome’s cargo is widely accepted as the main actor in cell regeneration, and their composition remains complex and impacted by cell networking and microenvironmental priming [36,37] giving rise to potential new markers involved in cell proliferation, healing, immunomodulation, and aging.

This review aims to highlight the relationships between ADSCs and their microenvironment through inducing specific biomolecules secretion within exosomes and involving different immune cell network by targeting specific molecular mechanisms and new targets for skin regeneration.

## 2. Skin: Anatomy and Physiology

Depending on people and age, skin thickness varies and averages between 0.05 to 2 mm. It’s consisted of three layers: the epidermis, which is non-vascularized and stratified, underlying another layer composed of connective tissue called the dermis. The hypodermis is the subcutaneous adipose tissue including the adnexal structure and supporting the dermis [38]. As an external barrier to factors penetration, an organized structure the stratum corneum formed by dead cells called corneocytes is disposed as bricks between multiple lipid bilayers and hold the structure defined as “brick and mortar” constituting the epidermis [39]. The generation of epidermis with its lipid-rich cornified layers is performed through progressive keratinocytes differentiation and proliferation from the basal stratum germinatevum to provide the outermost layer of newly committed cells [40]. To protect the skin against UV damages, melanocyte cells also present in the epidermis synthetize and transfer melanin pigment to mature keratinocytes. The epidermis also contains Merkle cells, dendritic cells, adipocytes, and Langerhans cells.

The dermis is composed mostly by fibroblasts, a mesenchymal cell type presenting elongation and shaped form and acting as skin scaffold to support the epidermis and other epithelial cells. Moreover, fibroblasts play a pivotal role in cutaneous strength and elasticity through secreting fibrous and elastic components constituting the Extracellular Matrix (ECM) [38]. ECM is a three-dimensional microenvironment composed of fibrous proteins, a ground substance and the vascular network closely related to collagen, elastin, and fibronectin fibers to provide supportive niche for epithelial cells and stem cells [41]. In human skin, the ECM accounts for 75% of the dry skin weight of collagen fibers type I, III, and V which confers elasticity and strength. Type I collagen is abundantly represented with 80–90% of the total collagen, and type III is 8 to 12%, while type V collagen remains minor [42]. Adding to its skin structural support, ECM play a critical role in regulating cell behavior in normal and healing settings [43]. Integrin cell surface were reported to mediate the molecular signaling pathways regulating cell orientation and turnover [44].

Immune cells reside in majority in the dermis and sometimes in the hypodermis and are represented by lymphocytes, macrophages, mast, and dendritic cells. The other adnexal supportive structures includes hair follicles, blood vessels, nerves and different glands including eccrine, sebaceous and apocrine glands and are located in the dermis and hypodermis.

The subcutaneous layer or hypodermis is composed of adipose tissue containing ADSCs, lymph, and blood vessels, and secrete a wide panel of cytokines and chemokines whereby homeostasis, thermoregulation, immunomodulation, metabolism, and immune responses occurs. This secretome was reported to address the microenvironment of ADSCs and impact their exosomes secretion and composition, thus modulating skin cell behavior, melanin production and recently inducing skin rejuvenation [45,46,47,48,49].

Additionally, aging by photoaging and age-dependent of the skin represents a real challenge of the new ADSCs advancements. Skin aging is morphologically apparent through epidermal atrophy and wrinkles appearance, reduction in the dermal thickness associated to an ECM degradation and adnexal structures decrease in number and function. The skin enrichment in epithelial cells including, dermal fibroblasts (DF), Langerhans cells and melanocytes was decreased as well. ADSCs ability to replicate decreases with age resulting on senescent and non-dividing cells. Additionally, self-renewal and mitotic activity of ADSCs are reduced leading to the thickness of subcutaneous adipose tissue improved by the increasing senescence profile. ADSCs might behave differently according to the context of stimulation, to modulate the different mechanisms underlying skin cell regeneration.

## 3. ADSCs and Exosomes Pathway

Increasing evidences have shown that Mesenchymal Stem Cell (MSCs) and ADSCs release enriched exosomes as actors in cell-to-cell and intracellular communications through transporting a wide range of proteins, lipids, and nucleic acids in normal and pathological processes. They are orchestrated through a Syntenin pathway, the endosomal-sorting complex required for transport ESCRT-dependent and two other ESCRT-independent pathways (Tetraspanins and Ceramide) [37]. Invagination of plasma membrane (PM) form endosomes by fusion of several primary vesicles. They mature during their intracellular trafficking from the PM to the cell center, leading to overall changes in their composition of lipids and proteins. Exosomes exhibit the first variability due to the membrane content of cell of origin [50]. Even though, their cargo is closely dependent on the cell type and their microenvironment. Exosomes are reported to exert similar effects as their cells of origin and ensure parental cell communications by releasing their cargo into the target cells or through binding by their membrane receptor to specific ligands in target cell.

**Figure 1:**
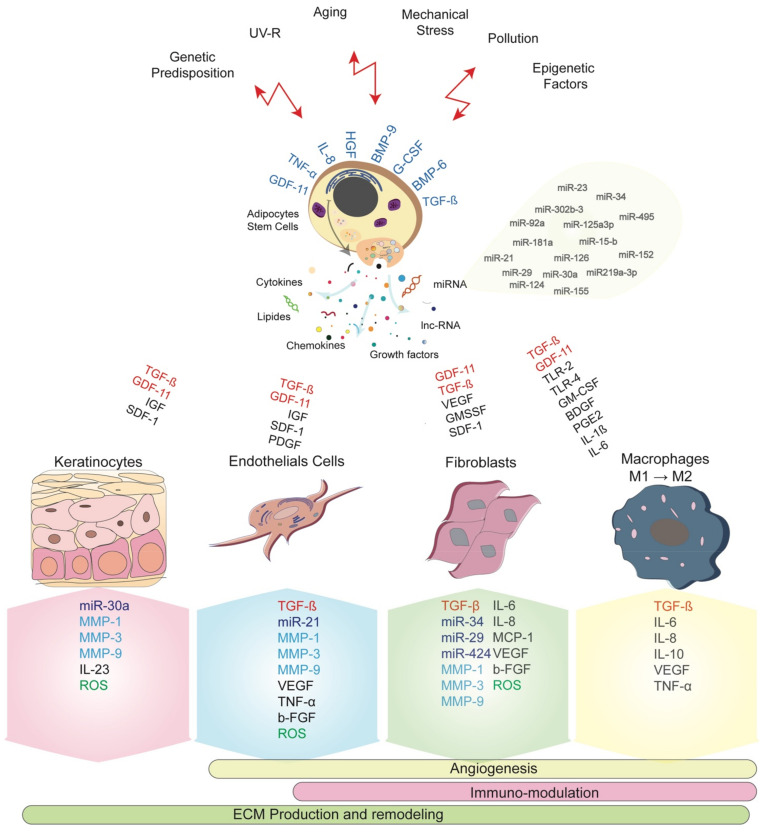
ADSCs within their network and exosomes-derived biomolecules involved in the different biological processes associated to skin aging and regeneration. ADSCs microenvironment is impacted by their cellular network and is closely dependent of its enrichment of skin and immune cells and their ability to secrete adequate and sustainable quantities of growth factors, cytokines, miRNAs and other transcriptional factors. Specific biomarkers are identified and might serve as new parameters for skin immunomodulation during skin regeneration and aging strategies.

Exosomes can be generated during stem cell expansion culture and similarly from waste products of large-scale biotechnological culture media [37,51,52]. However, the long-term culture expansions were largely reported to impact stem cell characteristics in terms of proliferation and differentiation ability, their gene expression profile and exosomes secretion [37,53]. Conditioned-media and stromal vascular fraction have been reported to auto-induce ADSCs and target skin cells represented by DF, melanocytes, keratinocytes, and dermal microvascular endothelial cells [19,54,55]. The Figure 1 summarizes the main interactions between ADSCs network and biomolecules secretion. Moreover, ADSCs present great ability in migration leading to their rapid recruitment into wounded sites where they secrete their enriched exosomes in response to the local damage or undergo the process of cell differentiation towards various skin cell components.

Exosomes-derived ADSCs are composed of transforming growth factors-β (TGF-β), Growth Differentiation Factor 11 (GDF11), Interleukin-6 (IL-6), IL-10, IL-8, IL-1β, Toll-like Receptor 2 (TLR2), TLR4 and Tumor Necrosis Factor (TNF)-α, Basic-Fibroblast Growth Factor (b-FGF), Granulocyte Monocyte-Colony stimulating factor (GM-CSF), Wingless 10b (Wnt10b), Stromal-Derived Factor-1 (SDF-1), Insulin-like Growth Factor (IGF), Hepatocyte Growth Factor (HGF) and Granulocyte-Colony Stimulating Factor (G-CSF) [35,56,57]. These factors are reported in skin and different organs functions [19,53,58,59,60,61,62].

Exosomes derived from adipose tissue-derived mesenchymal stem cells (AD-MSCs) have immunomodulatory effects of T-cell inflammatory response in autoimmune diabetes type 1 [63]. These authors have demonstrated the increase in T cell regulator with a decrease in proinflammatory IL-17 and interferon simultaneously to the increase in IL-4 and IL-10 levels. These findings were in concordance with the amelioration of the autoimmune reaction in Trastuzumab emtansine (TDM1) mice model and the glucose levels. ADSC-derived exosomes activated M2 macrophage polarization and reduced inflammation in obese mice [64]. ADSCs derived exosomes were also suggested as therapeutic option of the atopic dermatitis [65]. In mice model, these exosomes increased the number of neutrophils while reducing that of circulating eosinophils and the skin infiltering mast cells, CD86+ and CD206+ cells. Additionally, mRNAs of IL-4, IL-23, IL-31, and TNF-α were significantly reduced. ADSCs derived exosomes rather than the MSCs or their conditioned media improved neutrophils viability and functions and seems to be more beneficial in infections and immunodeficiency diseases [66]. Many advancements have recently paved the way to animal studies to investigate the role of ADSCs derived exosomes or from other sources as drug delivery systems [67]. Engineering approaches are performed to ensure bioavailability of safe and efficient ADSCs and bone marrow derived exosomes for immunomodulatory activity [68]. Recently, ADSCs derived exosomes rises great promises and hopes in treating pneumonia thanks to their broad pharmacological effects. A clinical trial is ongoing to shorten and inhibit the cytokine storm associated to the COVID-19 patients presenting acute respiratory distress symptom by secreting anti-inflammatory cytokines and stimulating immune cells enabling pneumonia treatment (http://clinicaltrials.gov).

In case of aging, ADSCs have proven their efficiency in reducing wrinkles and enhancing dermal thickness more likely by interacting with DF and favoring angiogenesis [35,56,69]. Factors as b-FGF, Vascular Endothelial Growth Factor (VEGF), TGF-β, platelet-derived growth factor (PDGF) and HGF are demonstrated to promote new blood vessels associated to the proliferative phase of wound healing by promoting cell differentiation into endothelial progenitor cells [58,70]. Actually, other cytokines and micro-RNA (miRNAs) are expected to play a key role in skin tissue regeneration such as Bone Morphogenic Protein (BMP)6, BMP9, IL-1, miR-21, miR-23a, and miR-124 by targeting DF, keratinocytes, and especially immune cells [24,33]. An immunosuppressive and anti-inflammatory role has been attributed to a number of these miRNAs [71].

Aging is another parameter impacting the immunomodulation effect of ADSCs. Accumulation of senescent cells increases the secretion of pro-inflammatory factors including IL-8, IL-6, and TNF-α associated with chronic inflammation [60,72]. We can consider that ADSCs are likely the main actor involved in skin regeneration during aging and wound healing. Their ability to proliferate, differentiate into skin cells, modulate the duration of the inflammatory phase and the re-epithelialization is potentially dependent on their exosomes composition and their cellular network within skin layers. When administrated in aged dermis, dermal thickness, as well as skin texture and wrinkles, were found improved 8 weeks after treatment [73]. The exosomes released in their secretome or conditioned media improve recipient cells secretion of ECM proteins including collagen and elastin deposition [25,26,59,74,75]. Accordingly, fibroblasts proliferation and migration increased and apoptosis delayed leading to a significant reversal of the aging process and the associated-skin symptoms [55,76].

## 4. Endogenous Messengers Associated to Skin Aging

It’s widely accepted that intrinsic signaling such as epigenetic factors and genetic predispositions and extrinsic factors such as ultraviolet (UV) radiations, water and air pollution are associated to aging by impairing skin integrity and youth. Additionally, UV or infrared irradiations are expected to be more cell damaging than intrinsic factors. More DNA damages are observed in skin cells following irradiations [47,77]. Accordingly, elasticity loss, wrinkles appearance, pigmentation dysfunction, or hyperkeratosis are reflecting the visual symptoms of aging as a result of the progressive dermis atrophy. These manifestations rely on the impairment of cell senescence, a multifactorial process leading to the loss of skin integrity. Collagen production was found decreases and its degradation increased giving rise to a quantitative and structural change in collagen fibers and the dermis structure [78,79,80].

### 4.1. MMP Pathway

During aging, DF, keratinocytes and although endothelial cells secrete ubiquitous endopeptidases having the ability to degrade the ECM proteins called Matrix Metalloproteinase (MMP). The major protease present in human skin MMP-1 degrades the collagen fibers type I and III, followed by MMP-3 and MMP-9 [81]. The process of degradation is regulated by the specific tissue, Inhibitors of Metalloproteinases (TIMPs) where their increase in levels during aging unbalances the intrinsic collagen level and distribution and accelerating skin aging. In photoaging, activation of MMPs-2, -3, -9, -12, and -13 is responsible for the degradation and disorganization of elastic fiber [79,82,83]. In addition to MMP1 and MMP2 expression, upregulation of α-Smooth Muscle Actin (α-SMA) was related to a fibroblast senescence phenotype following ultraviolet A (UVA) treatment [55]. nevertheless, Patel et al. have first suggested that MMP and TIMPs considered as a ratio MMP–TIMPs could reflect wound healing and aging [84]. Consequently, the increasing MMPs levels and decreasing TIMPs might be informative on the regenerative capacity of skin cells and the supportive role of the ECM (Figure 1).

### 4.2. Reactive Oxygen Species (ROS) Pathway

ROS is the most important type of free radicals produced as a result of the imbalance between oxidants and antioxidants, generated under physiological and pathological situations, and called oxidative stress. Free radicals with unpaired high reactivity electrons are produced through reduction-oxidation reactions (Redox). Different findings have reported that ADSCs functional properties were impacted by the redox mechanism during wound healing and aging [69,85]. Behind the increase in MMP levels, skin cells also generate ROS (Figure 1). The transcriptional factors activator protein 1 (AP-1) and Nuclear Factor-kB (NF-kB) activate the mitogen-activated protein kinase (MAPK) family and then induce upregulation of MMP in keratinocytes and DF [86,87,88].

During aging, increased ADSCs senescence and oxidative stress lead to an increase in exosomes release [85]. However, the exosome’s composition might act as oxidant or antioxidant factors depending on cell conditions and triggering redox mechanisms regulation. Adding to the fact that senescence is accompanied by enhanced ROS production, its prolonged release in the skin promote chronic inflammation probably through amplifying the inflammatory injury [89]. In osteoarthritis, ADSCs derived exosomes lead to the pro-inflammatory mediators reduction (TNF-α, Prostaglandin E2 (PGE2), IL-6 and Nitric oxide (NO) with an increase in anti-inflammatory cytokines, such as IL-10 [90]. Dysfunctions of mitochondrial electron transport chains and a decrease in mitochondrial activity were reported during aging, causing a higher ROS production [47,91,92]. Additionally, the accumulation of ROS induced oxidative damages of structural lipoproteins and proteins leading to cell senescence. On the other side, the decline of replicative capacity due to DNA damage such as DNA methylation, chromatin architecture change, histone deacetylation and gene expression change might also result in senescence through compromising the intended cellular function. Likewise, the decrease in DF size and spreading observed during progressive ECM degradation is correlated to the increase in mitochondrial ROS generation simultaneously to their impaired attachment du probably to the involvement of MMP production [86,93,94].

### 4.3. GDF11 Pathway

In human, GDF11 is associated to age-related diseases, and its serum level reflects the physiology of aging [95,96]. In many human organs, its circulating level has been related to aging [19,97,98,99]. This factor has proven efficacy in antagonizing aging in a specific manner [100] and has remarkably improved myocardial hypertrophy and inflammation [76,77,101].

Different in vitro and in vivo studies have demonstrated that ADSCs-conditioned media stimulated the rejuvenation of human skin through reducing wrinkles and improving skin elasticity in a GDF11-dependent manner [102,103,104]. Similarly, ADSCs extract activated DF and keratinocytes leading to their proliferation and migration into damaged sites [105]. Moreover, dermal density was increased and an anti-wrinkle effect observed after using umbilical cord-MSCs conditioned-media in vivo [19]. Interestingly, ADSCs from young donors produce more GDF11, and keratinocyte stem cells are more highly proliferative than cells from aged donors [106]. Behind using platelets rich plasma (PRP) in skin therapeutic purposes, anti-wrinkles and anti-aging aspects observed are likely related to its extremely higher concentrations of GDF11 [107].

Additionally, GDF11 expression and activity were reduced in adult DF compared to the neonatal ones [108]. Nevertheless, these cells play a crucial role in skin regeneration through activating the GDF11 mechanism in both neonatal and adult cells. Interestingly, MSCs derived from the placenta and umbilical cord blood improve fibroblasts plasticity [109] probably by the release of GDF11 and the stimulation of the skin rejuvenation [110]. GDF11 was shown to activated fibroblasts and increase ECM proteins production and especially collagen I and III and fibronectin [19].

Many evidences are arguing on the pronouncing effect of GDF11 on the expression of different genes belonging to different skin function (Figure 1). Recombinant (r) GDF11 was found to increase the expression of genes related to ECM production such as Collagen (COL)1A1, COL6A6, COL14A1, TGFBR3, ELN and HAS1. Genes involved in skin barrier function (Adipose tissue Lipoxygenase ALOX12, ALOX12B, ALOXE3, DSGI, DSP), cell proliferation, and turnover (enhancer of Zeste homolog 2 EZH2, EZH1, heparin-binding EGF-like growth factor HBEGF, Kallikrein-related peptidase KLK7, KRT6B) were improved in the same way [54].

Moreover, by increasing collagenase MMP-9 secretion, recombinant (r)GDF11 may contribute to matrix remodeling by interacting MMP-9 with TGF-β1 relaying on skin wound closure [111,112]. These cell interactions confirm the involvement of the TGF-β and GDF11 mechanisms used by ADSCs in managing the aging process [54,108,113]. After binding to their transmembrane specific receptors Activin IIB receptor (ActIIBR) and TGF-βR respectively, both factors acted through the TGF-β Smad pathway (R-Smad2/3) and R-Smad 1/5/8/). The intracellular signal transduction cascade involved the phosphatidilinositol3-knase (PI3K)/protein kinase B (AKT) pathway (PI3K/AKT) and targeted specific gene expression [28,54,99].

On the other hand, positive effects on skin vasculature, density, integrity, strength, and wrinkles reduction have been reported after using rGDF11 [54] likely by crosstalking between DF, ADSCs, keratinocytes, and endothelial cells. The improvement of skin microvasculature impaired during aging is realized through proliferation and differentiation of endothelial progenitor cells [114]. On one side, DF secretes TGF-β, VEGF, and b-FGF leading to angiogenesis [70,115], and, on the other side, ADSCs accelerated neovascularization through the expression of hypoxia-inducible factor (HIF)-1α [116] by regulating VEGF gene expression in endothelial cells [117].

Additional indicators have been associated with epidermal biology. The transcription factors such as P63 (P53 family) and P16^INK4a^ interfere with keratinocytes senescence. Indeed, P16^INK4a^ positive cells increased with age-dependent aging in human dermis and epidermis while P63 expression was reduced [118,119,120]. In the same way, Notch and Wnt/β-catenin were reported in epidermal lineage commitment [120,121].

## 5. ADSCs Network in Skin Repair and Regeneration

Increasing evidences showed the cellular and molecular ADSCs involvement in maintaining skin homeostasis. During normal development, these resident cells ensure skin regeneration and repair after injury [46] (Figure 1). ADSCs are located in the basal layer where they are responsible for recruiting and directing mature differentiated cells (keratinocytes) to the outer of the epidermis. In the epidermis, these stem cells undergo self-renewal and a sustainable production of transient amplifying cells following a hierarchic gradient to ensure skin regeneration [122].

To maintain the microenvironment propitious to cell turnover, epidermal cells are closely interacting with each other. Adding to their aptitude to differentiate into DF, keratinocytes and probably melanocytes, interactions of ADSCs with these cells is a part of normal skin function where ECM secretion provided a supportive microenvironment necessary for the maintenance of the stem cell niche [123]. Hur et al. have also demonstrated these ADSCs interactions when skin fibroblasts cell line HS27 activate ADSCs to differentiate into fibroblast-like cells highly expressing Heat shock protein-**47** (HSP47), vimentin, and desmin mRNA level [110].

Moreover, ADCSs secrete exosomes to modulate the homeostasis of the skin microenvironment by releasing and regulating the genetic expression of the different actors implicated in neo-angiogenesis, cell proliferation and differentiation, and cell migration [25,26,59].

Either secreted by ADSCs, TGF-β is also released by DF, macrophages, to amplify angiogenesis and migration of ADSCs and epithelial cells by stimulating the SMAD2/3 pathway and increasing the expression of CXCR-4 receptor of SDF-1. In an in vitro and recent study, ADSCs migrate into damaged sites and were recruited using the SDF-1/CXCR-4 axis and the intracellular Janus kinase (Jak)/AKt regulation pathway [19]. Likewise, GDF11 activate migration of DF and keratinocytes into wounded sites. Using the same SMAD2/3/pathway, both GDF11 and TGF-β stimulate the migration of skin endothelial cells to improve angiogenesis, suggesting that GDF11 might activate similar pathway in DF and keratinocytes to ensure cell migration and wound repair. Other mechanisms, such as Jerky gene (JRK), and Extracellular signal-Regulated kinase (ERK) signaling activated by activin B were reported and leaded to actin stress fiber formation involved in cell migration [9]. Additionally, activin B promotes ADSC migration by enhancing α-SMA expression and stress fiber formation.

The participation of ADSCs in ECM production is highly expected. ECM accumulation plays a pivotal role in cell migration and angiogenesis by amplifying the secretion of the involved growth factors. At the other side, macrophages secrete collagen I and III, elastin, and fibronectin and activate DF and endothelial cells to proliferate and migrate. TGF-β interactions with ADSCs and DF amplify collagen production and inhibits ECM degradation by increasing TIMPs secretion and their binding to MMPs, thus favoring the remodeling phase [124]. During this phase, adipose tissue secretome stimulates fibroblasts to express fibronectin and migrate by increasing N-cadherin and CD44 adhesion molecules [125]. A combination of activin B and ADSCs ensure rapid wound closure and accelerate epithelialization by promoting fibroblasts and keratinocytes proliferation [9]. The exclusive αβ6 integrin expressed by epithelial cells is involved in the regeneration of basement membrane zone during wound repair [126].

In another point of view, interactions of ADSCs and microvascular endothelial cells play a critical role in skin cell proliferation and regeneration by providing monocyte chemoattractant protein-1 (MCP-1), IL-6, IL-8 and VEGF to modulate inflammation and angiogenesis [127,128]. Recently, IL-23 expression in keratinocytes seemed to be associated with GDF11 limiting thus the immune cell infiltration and epidermal thickening [129], while IL-1β expression was decreased [54]. Other findings suggested that inhibiting TNF-α release by macrophages after activation by the NF-KB signaling pathway is more susceptible to decrease inflammation [130,131]. Otherwise, NF-kB pathway was targeted by GDF11 and decreased leading to protection against apoptosis.

In normal conditions, human serum and platelets actively stimulate ADSCs to proliferate and differentiate. In wounded tissues, platelets secrete PDGF, IL-6 and IL-8 and activate stem cells to initiate the inflammatory phase leading to migration of neutrophils and macrophages to the wounded site [132]. Secretion of TGF-β activates monocytes into macrophages. Additionally, ADSCs secrete TNF-α, prostaglandin E2 (PGE2) and GDF11 to amplify the proinflammatory responses and anti-inflammatory cytokines secretion through polarization of macrophages from M1 to M2.

## 6. Immunoregulatory Parameters in the Skin: Emerging New Biomarkers

Increasing evidence suggests that the immunomodulation capacity of ADSCs, and their presence in the epidermal layer, lead them to play a pivotal role in skin immunological functions at both physiologic and injured settings (Figure 1). The ADSCs’ migration to the injured site holds great interest, their immune profile and their potential shift towards an anti-inflammatory phenotype is critical to the proliferation and remodeling stages of healing [133,134,135]. Moreover, the cytokine profile of T and B lymphocytes and dendritic cells was influenced by ADSCs accelerating the inflammatory phases and initiating the proliferation and remodeling phases in chronic wounds [136].

### 6.1. TGF-β–GDF11 Ratio

Different findings reported that TGF-β was involved in skin regeneration more than participation in aging and is considered as a tool key in the regulation of wound healing [28,137]. Through their immunoregulating ability, GDF11 and TGF-β also take part in skin inflammatory process during wound healing and skin aging or inflamm-aging by downregulating pro-inflammatory cytokine genes expression [60,72]. Otherwise, TGF-β is responsible for the loss of the immune antimicrobial function of DF during aging [138]. Regarding TGF-β and GDF11 secretion levels and skin regeneration and youth characteristics during aging [35,61,100], an expected ratio of TGF-β–GDF11 might be considered and regulated in a spatio-temporal manner, thus balancing the whole cellular and molecular mechanisms associated to regeneration or rejuvenation. This ratio kinetic might be considered as a biomarker of the functional behavior of skin ADSCs and cell composition and enrichment in immune cells. These regulating aspects must drive specific attention as a key target to achieve antiaging processes during wound healing. More investigations on GDF11 and TGF-β levels and their relationships in skin function and behavior are needed, opening thus the way to new potential strategies in skin inflamm-aging treatment.

Even though, ADSCs also regulate the fate of melanocytes by modulating enzyme-producing melanin activity. They increase their TGF-β secretion and induced melanocytes to downregulate the expression of melanogenic enzymes and prevent site-specific pigmentation in reconstructed skin grafts. These interactions might have applications in modulating melanin synthesis through TGF-β and impacting the skin whitening [139]. Additionally, DF impacted skin pigmentation by regulating melanocytes maturation and melanin-producing enzymes by increasing TGF-β secretion [113]. These findings altogether suggested that the cellular composition of the dermis might control the production of mature melanocytes and melanin transfer to keratinocytes. Recombinant GDF11 was also shown to significantly reduce melanin production in melanocytes and in 3D skin equivalents [54]. Otherwise, ROS accumulation associated to aging and the replicative capacity, is responsible for the occurrence of vitiligo and its progression [140]. These reports might be useful in considering the potential use of GDF11 and TGF-β ratio in different skin types and color. This ratio could reflect the balance between skin pigmentation and skin youth and considered as sustainable mechanisms governing skin biology and function. Moreover, we can postulate that white skin is expected to be more youthful and contain higher amounts of GDF11 leading to less cell apoptosis and senescence events. These observations need further advancements in the future to better understand the paradigms triggering the mechanisms related to skin aging and pigmentation.

The miRNA paradigm has triggered different interest since their description as major actors in intracellular and intercellular signaling and homeostasis, cell senescence, and aging [141,142,143,144] especially in skin biology [142,145,146,147,148]. Skin cell components including ADSCs crosstalk altogether through transmitting these miRNAs as pleiotropic messages [47,48,49,148,149]. MiRNAs such as miR-152, miR-181-a, -92a, -15b, -125a-3p, -219a-3p correlated with skin aging and were associated with fibroblasts senescence [142,143,150].

The screening miRNA gene expression profile has identified the MiR-495 as a therapeutic target for hypertrophic scars [145]. MiR-34 family (miR-34b-5p), miRNA-29 family, and miRNA-424 were differentially expressed in aged dermis compared to the younger ones, and these miRNA are involved in cell adhesion, collagen synthesis through different signaling pathways such as P16 in regulating fibroblasts senescence and in photoaging [147,151]. Other miRNAs have been related to fibroblast senescence, miR-302b-3p inhibitor might hamper fibroblast senescence and contribute Sirt1 expression targeting thus directly c-jun nuclear kinase2 (JNK2) gene [146]. In addition, miR-30a targeted genes identified in aged keratinocytes and relied on their differentiation and apoptosis profile such as LOX, isocitrate dehydrogenase1 (IDH1), and AVEN [152]. MiRNAs are also targeted by the intracellular signaling regulating aging, thereby ROS and cytosolic calcium modulate their expression under different conditions including inflammatory-associated diseases and UV exposure [141,151].

Recent studies have shown that miRNAs also play a crucial role in regulating the immunomodulatory activity of stem cells [153,154]. MiRNA-126 and miRNA-23 overexpression, miRNA-21, and miRNA-155 downregulation are involved in the inflammatory mechanisms through the expression of PI3K\AKT1\NF-KB genes [154]. This signaling pathway seems likely to be involved by the TGF-β superfamily including TGF-β and GDF11.

Other biological mechanisms included in wound repair are modulated by miRNAs. Derived from umbilical cord and ADSCs, miRNA-21 promoted vascularization of endothelial cells and angiogenesis by upregulating SDF-1, HIF-1, VEGF, p-Akt, p-ERK1/2 and downregulating Phosphate and tension homolog (PTEN) and sprout homolog 1 (SPRY1) [27,33]. Enriched miRNA-21-3p exosomes led to accelerated re-epithelialization, reduced scar widths in addition to increasing angiogenesis, suggesting that this miRNA could be used as a healing strategy in skin pathology and other soft tissue [27].

### 6.2. M1 and M2 Macrophages Balance

Macrophages appeared to be closely associated to ADSCs and are involved in many biological processes by mediating immune cells to skin inflammation. In wound healing, macrophages were recruited and underwent polarization from M1 to M2, by similarity, macrophages infiltrating in vivo vascularized human dermo-epidermal skin substitutes transient from the CD68+ nitric oxide synthase iNOS+ M1 profile to the CD68+ CD206+ M2 during the phases of healing [155,156]. Nevertheless, accumulation of senescent cells in the skin produced pro-inflammatory factors such as TNF-α, IL-6, and IL-8, thus changing the inflammatory profile of the associated microenvironment as in aged tissues [157]. The pro-inflammatory cytokine IL-1β highly supports skin inflamm-aging. ADSCs and microvascular endothelial cells increased secretion of MCP-1, IL-6, and IL-8 to modulate skin inflammation [128]. Moreover, skin autoimmune inflammatory diseases were associated to infiltering activated macrophages [129,153,158].

TGF-β is highly involved during the whole process of wound healing [16]. This factor activates the secretion of ECM proteins and M2 macrophages polarization leading to angiogenesis, DF proliferation, cell migration and re-epithelialization. Koivisto et al. have demonstrated that TGF-β modulates the innate immune surveillance in the skin as a result of the integrin αβ6 secretion by epithelial cells [126]. Additionally, the collagen triple helix repeat containing one protein contribute to the healing process via increasing TGF-β expression level and M2 macrophages recruitment [159]. Similarly, IL-1β and IL-6 increases macrophages recruitment, and their polarization from M1 to M2 [155] leading to the secretion of anti-inflammatory cytokines [156]. Macrophages activation appears to play a pivotal role in the secretion of ECM proteins, angiogenesis, cell proliferation, migration, and re-epithelialization.

There are evidences that ADSCs can differentiate between the M1 pro-inflammatory and M2 anti-inflammatory macrophages phenotype. Priming ADSCs by using inflammatory agents induces the activation of macrophages from M1 to M2 profile and the activation of Treg cells [31,156,160]. Analyzing the expression levels of the CD14, CD64, CD80, CD163, and CD200R in macrophages might lead to evaluate their suppressive effect and consequently their response to the immunomodulating effect of ADSCs. Assessment of M1–M2 ratio or level could be of interest as a healing indicator providing information guiding the improvement of new strategies of anti-inflammatory cytokines administration, especially in non-healing skin diseases.

## 7. Conclusions

ADSCs act through their exosomes to improve and induce tissue repair. They ensure ADSCs interconnections with skin components and microenvironment to provide repair and regeneration, thus opening the way for the new cell-free therapy [75,161]. These cells have a protective and antiaging effects on DF by preventing their oxidative stress and increasing their superoxide dismutase and glutathione peroxidase activities [69]. In wound defects, ADSCs migrate rapidly into injured sites where they differentiate into skin cell components. However, ADSCs take part of the whole process of wound healing through autocrine and paracrine pathways. Otherwise, the increased paracrine senescent secretome of ADSCs during aging might reinforce inflammation within their microenvironment [157].

The other cells considered as the most immune cells involved in the innate immune skin surveillance are macrophages. Recently they are described as the key regulator of skin immunomodulation as they infiltrate skin layers in inflammation-associated diseases and during aging. Interactions between resident skin ADSCs and local macrophages lead to changes in the immunological phenotype of both cells which is reflected by the polarization from M1 pro-inflammatory profile to M2 anti-inflammatory profile, the suppression of Th cells, and the increase in Treg cell activation [31]. ADSCs’ changes are also relevant through the secretion of anti-inflammatory cytokines and the increase in the expression of genes involved in skin homeostasis [54].

Moreover, ADSCs’ functions are modulated by the composition of their microenvironment on immune cells and change their behavior in response to the nature of biomolecules [28,46,162]. This conditioned media might induce macrophages to secrete IL-10 and TNF-α anti-inflammatory cytokines, and on the other side to decrease the secretion of the pro-inflammatory cytokines [19,163].

Given their direct skin cell contact, ADSCs orchestrate and accelerate the mechanisms supporting their differentiation into DF and keratinocytes, melanin production, neo-angiogenesis, and increase in capillary density and finally the re-epithelialization necessary for wound closure [28]. The reciprocal interactions between ADSCs and macrophages let us suggest that dermal composition in ADSCs and macrophages is the key regulator of the skin immune profile [28,30]. Their respective number would impact the inflammatory status of the skin and consequently skin cell regeneration and function in both normal and wounded conditions, by modulating the fate of ADSCs [31]. Indeed, recent work has confirmed that the immunomodulatory effect of ADSCs is significantly dose dependent in an animal model [164]. Moreover, the clinical efficiency of ADSCs remains dependent on the number of cells injected, their route of administration, and their location of origin as well. Visceral ADSCs secrete higher quantities of inflammatory cytokines IL-6, IL-8, and TNF-α when compared to the subcutaneous ones [165]. ADSCs preconditioning with inflammatory or pro-inflammatory cytokines increase their survival and improved their responses to cancer and inflammation [32,153,165,166,167]. This epigenetically modification of the microenvironment of ADSCs might address cell ability to differentiate and migrate, and to restore cellular defects associated to aging.

Another critical point to consider is that patient-associated factors lead to inherent variability relative to ADSCs viability, self-renewal ability, differentiation potency, and their exosomes profile containing immunomodulatory mediators. In those collected from donors presenting medical histories, such as breast cancer, Crohn’s diseases, or other inflammatory diseases, ADSCs present an inflammation induced secretome associated with their self-renewal capacity [77,168].

Despite the limitations reported in the ADSCs use, ADSCs-derived exosomes offer a new therapeutic potential improvement by preventing some of these limitations [28,168]. Exosomes cannot divide and induce immunogenicity which is due to the absence of immunogenic components. Correspondingly their systemic use would be more beneficial to ensure the maximum efficiency in injured sites where administrated ADSCs could move to off-target damaged sites for cell regeneration. Additionally, by identifying and providing the therapeutic doses to administer regarding the immunomodulation related physiopathology, their composition might be optimized and enriched by specific biomolecules (cytokines or miRNA) through genome editing technology. Exosomes biogenesis and release enhancement by ADSCs is another issue paving the way to their enrichment for free-cell therapy. These considerations demonstrate the potential of ADSCs derived exosomes in treating skin inflammations associated to wound healing and skin aging.

## Abbreviations

ADSCs	adipose derived stem cells
DF	dermal fibroblast
ECM	extracellular matrix
GDF 11	growth differentiation factor 11
IL	Interleukin
MAPK	mitogen-activated protein kinase
MiRNA	micro-RNA
MMP	matrix metalloproteinase
MSCs	mesenchymal stem cells
PM	plasma membrane
PDGF	platelet derived growth factor
ROS	reactive oxygen species
TGF-β	transforming growth factors-β
TNF-α	tumor necrosis factor-α
TIMPs	tissue inhibitors of metalloproteinases
VEGF	vascular endothelial growth factor
